# Decentralized Learning Framework of Meta-Survival Analysis for Developing Robust Prognostic Signatures

**DOI:** 10.1200/CCI.17.00077

**Published:** 2017-11-01

**Authors:** Yi Cui, Bailiang Li, Ruijiang Li

**Affiliations:** **Yi Cui**, **Bailiang Li**, and **Ruijiang Li**, Stanford University School of Medicine, Stanford, CA; **Yi Cui**, Global Institution for Collaborative Research and Education, Hokkaido University, Sapporo, Japan.

## Abstract

**Purpose:**

A significant hurdle in developing reliable gene expression–based prognostic models has been the limited sample size, which can cause overfitting and false discovery. Combining data from multiple studies can enhance statistical power and reduce spurious findings, but how to address the biologic heterogeneity across different datasets remains a major challenge. Better meta-survival analysis approaches are needed.

**Material and Methods:**

We presented a decentralized learning framework for meta-survival analysis without the need for data aggregation. Our method consisted of a series of proposals that together alleviated the influence of data heterogeneity and improved the performance of survival prediction. First, we transformed the gene expression profile of every sample into normalized percentile ranks to obtain platform-agnostic features. Second, we used Stouffer’s meta-z approach in combination with Harrell’s concordance index to prioritize and select genes to be included in the model. Third, we used survival discordance as a scale-independent model loss function. Instead of generating a merged dataset and training the model therein, we avoided comparing patients across datasets and individually evaluated the loss function on each dataset. Finally, we optimized the model by minimizing the joint loss function.

**Results:**

Through comprehensive evaluation on 31 public microarray datasets containing 6,724 samples of several cancer types, we demonstrated that the proposed method has outperformed (1) single prognostic genes identified using conventional meta-analysis, (2) multigene signatures trained on single datasets, (3) multigene signatures trained on merged datasets as well as by other existing meta-analysis methods, and (4) clinically applicable, established multigene signatures.

**Conclusion:**

The decentralized learning approach can be used to effectively perform meta-analysis of gene expression data and to develop robust multigene prognostic signatures.

## INTRODUCTION

Gene expression-based prognostic (survival) models can serve as useful biomarkers that guide clinical decision making for precision medicine.^1,2^ Although many of them have been proposed, many have failed to be validated on external independent datasets, and few have been incorporated into routine clinical practice.^3^ A major issue has been that the discovery of most models was developed on relatively small cohorts that usually came from a single institution. The limited sample size can cause overfitting and false discovery, which leads to spurious findings and overconfident results.^4^ Better approaches to developing robust prognostic models using gene expression data are needed.

Combining data from many studies—performing a meta-analysis—overcomes the limitations of small sample sizes by increasing statistical power and by allowing the robustness of findings to be assessed across multiple cohorts. Given the availability of large-scale public databases, such as the National Center for Biotechnology Inormation Gene Expression Omnibus (GEO), ArrayExpress (Cambridge, United Kingdom), and National Cancer Institute’s The Cancer Genome Atlas (TCGA), meta-analysis is becoming increasingly important for investigating high-throughput genomic and transcriptomic data.^5-7^ Previous meta-analytic studies in breast cancer,^8^ lung cancer,^9^ ovarian cancer,^10^ and pan-cancers^11^ only investigated single prognostic genes. However, a prognostic model, which integrates multiple genes synergistically interacting in biologic processes, holds the potential to further improve the prediction accuracy.^12^

The conventional method to train such a model is the Cox regression analysis,^13^ where the model parameters are obtained by maximizing the partial likelihood function. To avoid overfitting in the large-p-small-n scenario in genome-wide transcriptomic studies, the L^1^-regularized version of Cox regression is often used as well.^14^ Survival analysis has also been formulated as a ranking problem, where the concordance index (c-index) instead of the partial likelihood is maximized for model optimization.^15^ In addition, several methods have been proposed that are based on support vector machines,^16-18^ Bayesian methods,^19,20^ principle component analysis,^21^ area under the receiver operating characteristic curve optimization,^22^ or ensemble machines.^23^ However, existing methods are designed for model training using a single cohort. To our knowledge, there has been no previous systematic approach to training a prognostic model from multiple datasets by leveraging the power of the meta-analysis.

Nevertheless, to extract useful information from diverse data remains a daunting challenge given the profound biologic heterogeneity among datasets and technical biases across measurement platforms. One practical solution has been to apply batch-effect correction algorithms^24^ or feature transformation^25^ to the datasets and merge them together. However, this merging approach essentially requires a uniform or similar distribution of known prognostic factors (biologic, clinical, or demographic) across different cohorts. As a consequence, certain selection criteria have to be applied to carefully adjust for the population differences at the expense of reduced statistical power. Furthermore, when the meta-analysis involves cohorts receiving different therapies, or even distinct tissue of origins (eg, in pan-cancer studies), merging is questionable because it introduces confounding factors and because comparing the survival duration across cohorts no longer makes sense.

In this article, we present a novel decentralized learning framework of meta-survival analysis for training a prognostic model from multiple gene expression datasets without actually merging them. Such a strategy, along with the proposed feature transformation as well as carefully selected loss function, effectively overcomes the data heterogeneity stemming from both population and measurement biases.

## MATERIAL AND METHODS

### Gene Expression Data

We used the R package GEOquery to retrieve the processed expression data for the GEO datasets, with the exception of GEO datasets GSE32062, GSE17260, and those used in experiment 4. For GSE32062 and GSE17260, the processed data were z-score transformed across samples and lost the original ranking order. Therefore, their raw data were directly downloaded from GEO and transformed to percentile ranks without normalization. For the datasets in Experiment 4, we also downloaded the raw expression data but further performed normalization within each individual dataset by the robust multi-array average algorithm^26^ to best reproduce the cell cycle progression (CCP) signature as performed by the authors.

For the METABRIC dataset, the normalized expression data were obtained from SYNAPSE (Seattle, WA; www.synapse.org/#!Synapse:syn1688369/) with institutional review board approval. In addition, the processed data for E.MTAB.386 (transcription and microRNA profiling by array of human high-grade, late-stage serous ovarian cancers) and The Cancer Genome Atlas Ovarian Cancer were obtained from ArrayExpress (EMBL-EBI, Hinxton, United Kingdom; www.ebi.ac.uk/arrayexpress/experiments/E-MTAB-386/) and the University of California Santa Cruz Cancer Genomics Browser (version 2015),^27^ respectively.

### Preprocessing

For any gene corresponding to multiple probesets, the probeset with the largest mean expression was selected to represent that gene, because this leads to the one with the highest signal-to-noise ratio. For each of the experiments, only the genes commonly present in all the datasets involved were kept for subsequent analysis.

### Feature Transformation

For each sample, we applied a feature transformation method that transformed the expression profiles into normalized percentile ranks.^28^ Specifically, we ranked all the genes on the basis of their expression values and then divided these ranks by the total number of genes and used the normalized ranks as features. Because the ranks of the genes only depend on their relative abundancy in the transcriptome, this transformation maximally decouples the features from particular platforms or normalization algorithms and therefore allows the integration of data from various sources. Compared with quantile normalization^29,30^ where the result is dependent on the particular study cohort, percentile ranking is performed intrasample and produces strictly uniform distribution for the transformed features. We showed that the percentile rank-based gene signatures had a higher stability than raw expression-based signatures when evaluated on microarray and RNA-Sequencing platforms (Appendix). Another important advantage of ranking-based gene signatures is that they can be used in a truly individualized manner that facilitates their practical implementation.

### Gene Prioritization

After feature transformation, we prioritized the genes on the basis of Stouffer’s meta-z method.^31^ Specifically, for each gene, we calculated its z-score with respect to the survival in dataset k, z_k_, corresponding to the Harrell’s c-index test.^32,33^ The meta-z score was then given by $\text{z}=\sum_{1}^{\text{K}}{\text{z}}_{\text{k}}/\sqrt{\text{K}}$, where K is the total number of datasets and the meta–*P* value was Φ(z), where Φ(⋅) is the standard normal cumulative distribution function. We ranked the genes on the basis of their meta*–P* values and kept the top d genes for model training. We chose d o be the number of genes whose adjusted overall *P* values were smaller than .05 after Benjamini-Hochberg correction.^34^

### Development of Gene Signatures

We used the decentralized learning framework to develop multigene signatures by combining multiple datasets. Mathematically, this entails minimizing the joint loss function, $\text{loss}=\sum_{1}^{\text{K}}{\text{loss}}_{\text{k}}$ where loss_k_ is the loss of the model on dataset k. In particular, we aimed to build a linear model that minimizes the loss function, which is defined as the survival discordance (ie, in opposite direction of Harrell’s c-index) as follows:

$$\text{loss}\left(w\right)=\sum_{\text{k}=1}^{\text{K}}\sum_{\text{i}:\;{\text{c}}_{\text{i}}^{\left(\text{k}\right)}=1}\times [\sum_{\text{j}:\;{\text{y}}_{\text{j}}^{\left(\text{k}\right)}>{\text{y}}_{\text{i}}^{\left(\text{k}\right)}}1\left(w^{\text{T}}x_{\text{i}}^{\left(\text{k}\right)}-w^{\text{T}}x_{\text{j}}^{\left(\text{k}\right)}\right)]$$ (1)

where 1(⋅) is the Heaviside step function, y_i_ is the survival time, c_i_ is the censoring label, and $x_{\text{i}}^{\left(\text{k}\right)}$ is the feature vector (ie, normalized gene ranks) of the i-th sample in dataset k. Because of the nondifferentiability of the step function, we approximated it with the hinge function, such that the loss function becomes

$$\text{loss}\left(w\right)=\sum_{\text{k}=1}^{\text{K}}\sum_{\text{i}:{\text{c}}_{\text{i}}^{\left(\text{k}\right)}=1}\times [\sum_{\text{j}:{\text{y}}_{\text{j}}^{\left(\text{k}\right)}>{\text{y}}_{\text{i}}^{\left(\text{k}\right)}}\max\left(0,1+w^{\text{T}}x_{\text{i}}^{\left(\text{k}\right)}-w^{\text{T}}x_{\text{j}}^{\left(\text{k}\right)}\right)]$$ (2)

Because of the convexity of the hinge function, equation (2) is also convex. Therefore, minimization of the loss function can be achieved by plugging its gradient (more precisely, subgradient) to any convex optimization solver such as the quasi-Newton or Broyden-Fletcher-Goldfarb-Shanno methods. In the following equation, we provide an efficient algorithm to compute the subgradient of equation (2) with respect to **w**. Without loss of generality, we only derived the subgradient of the loss function for dataset k and for simplicity’s sake dropped the dataset index k, because the subgradient of the total loss is the sum of the subgradients of respective losses. According to equation (2), the subgradient of a given dataset is given by

$$\frac{\partial \text{loss}\left(w\right)}{\partial w}=\sum_{\text{i}:{\text{c}}_{\text{i}}=1}\sum_{\text{j}:{\text{y}}_{\text{j}}>{\text{y}}_{\text{i}}}\left(x_{\text{i}}-x_{\text{j}}\right)\cdot 1\left(1-\delta_{ji}\right)$$ (3)

where $\delta_{\text{ji}}=w^{\text{T}}x_{\text{j}}-w^{\text{T}}x_{\text{i}}$. Naïve evaluation of the above subgradient given **w** takes O(md) time, where m is the number of elements in the list $\text{E}=\left\{\left(\text{j,i}\right)|{\text{c}}_{\text{i}}=1,{\text{y}}_{\text{j}}>{\text{y}}_{\text{i}},1\leq \text{i},\text{j}\leq \text{n}\right\}$. However, a better implementation is to traverse E while maintaining an n × 1 vector **u** to keep track of how many times each feature vector **x**_i_ is selected based on the value of $\delta_{\text{ji}}$. More specifically, we initialized **u** = (0,0,…,0)^T^ and for each (j,i)∈ E, we updated ${\text{u}}_{\text{i}}\leftarrow {\text{u}}_{\text{i}}+1$ and ${\text{u}}_{\text{j}}\leftarrow {\text{u}}_{\text{j}}-1$ if $\delta_{\text{ji}}<1$. Then, after the traversal, the subgradient was simply **X**⋅**u**, where **X** = (**x**_1_,**x**_2_…,**x**_n_). This algorithm takes O(m + nd) time, which is faster than O(md) as in general m = O(n). The pseudo code for computing the loss function and its subgradient is summarized in algorithm 1.

Furthermore, to avoid overfitting, we regularized the joint loss with the ridge penalty. Therefore, the coefficient vector **w** was obtained by minimizing $\text{loss}\left(w\right)+\lambda {\left\|w\right\|}_{2}^{2}$. We performed cross-validation in a leave-one-dataset-out manner to select the penalty strength λ. That is, for a given λ, we trained a model on the basis of all the datasets but one and then computed the loss on the hold-out dataset. This procedure was repeated for each dataset, and the cumulated loss was computed. The penalty strength that yielded the minimum cumulated loss was selected. The final model was trained using all the datasets with the optimal penalty parameter.

Algorithm 1: compute loss function L and its subgradient **g**

Input:

$$\begin{array}{c} X=\left(x_{1},x_{2},\ldots \text{,}x_{\text{n}}\right)\text{,}\;w\text{,}\\ \;\text{E}=\left\{\left(\text{j},\text{i}\right)|{\text{c}}_{\text{i}}=1,{\text{y}}_{\text{j}}>{\text{y}}_{\text{i}},1\leq \text{i},\text{j}\leq \text{n}\right\} \end{array}$$

Procedure:

 L = 0
 **u** = (0,0,…,0)^T^
 **b** = **X**^T^**w**
 for each (j,i) in E do
  if b_j_-b_i_ < 1 then
   L←L + 1 + b_i_-b_j_
   u_i_←u_i_ + 1
   u_j_←u_j_-1
 **g** = **Xu**

Output:

 L, **g**

### Evaluation Criterion

Harrell’s c-index^32,33^ was used to evaluate and compare the predictive performance of the gene signatures. The c-index ranges from 0 to 1, with 1 being perfect prediction and 0 being the opposite. A c-index of 0.5 indicates random prediction.^32,33^ Here, c-index was chosen as the performance measure because it assesses whether a risk model can correctly rank the survival for every pair of patients. Therefore, it is a direct characterization of the overall prediction performance. C-index has been a popular choice for benchmarking survival predictors. For example, it is the adopted evaluation criterion in the DREAM (Dialogue for Reverse Engineering Assessments and Methods ) Breast Cancer Prognosis Challenge.^23^ This, in fact, is also why we aimed to minimize the survival discordance (ie, the opposite of c-index) as the loss function in our model. Interestingly, it has been shown that maximizing Cox’s partial likelihood function approximately maximizes c-index.^15^ However, as we have shown in algorithm 1, the hinge approximated survival discordance, and its gradient can be evaluated much more efficiently than the partial likelihood.

In addition, we evaluated the performance using the univariable Cox regression analysis. That is, after a survival model (gene signature) was trained on the training datasets, it was applied to the independent testing dataset to produce a risk score. This risk score was then analyzed using Cox regression to assess its correlation with survival in the testing cohort. Importantly, the risk score was treated as a continuous covariable instead of a binary one on the basis of a certain cutoff in the Cox regression, so that the performance was irrelevant to arbitrary cutoff choices. We compared the Cox *P* values of the gene signatures with a smaller value indicating better prediction.

## RESULTS

### Overview of the Proposed Method

**Fig 1.**
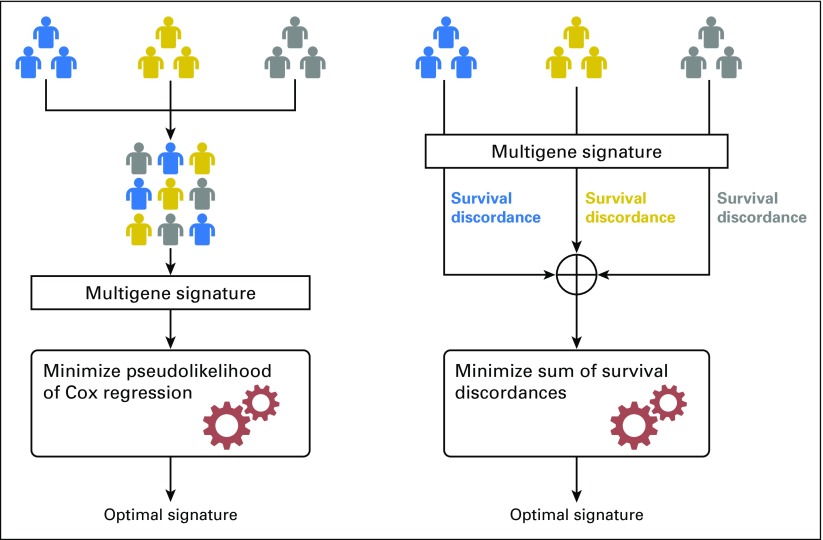
Schematic overview of (A) the proposed method on the basis of decentralized learning versus (B) the method on the basis of data merging for developing multigene signatures using meta-analysis.

To our knowledge, we are introducing a new approach to conducting meta-analysis to develop robust multigene signatures. Compared with a straightforward merging approach,^35^ our approach allows training a prognostic signature by integrating information from multiple datasets without actually merging them (Fig 1). This is achieved by using a decentralized learning strategy, where the loss functions (survival discordance) are computed within each dataset and then summed into an overall loss function. This approach, as opposed to simple aggregation and merging, eliminates the need to compare between patients who come from different populations and may have distinct clinical characteristics or have undergone different treatment regimens. As such, the gene signature built by decentralized learning will only reveal the common underlying genetic drivers across populations rather than being influenced by population-specific biases.

### Experimental Design

To demonstrate the effectiveness of our approach, we performed four different experiments as described below (Table 1). Altogether, we analyzed 31 public microarray datasets encompassing 6,724 gene expression profiles of patients with cancer.

**Table 1.**
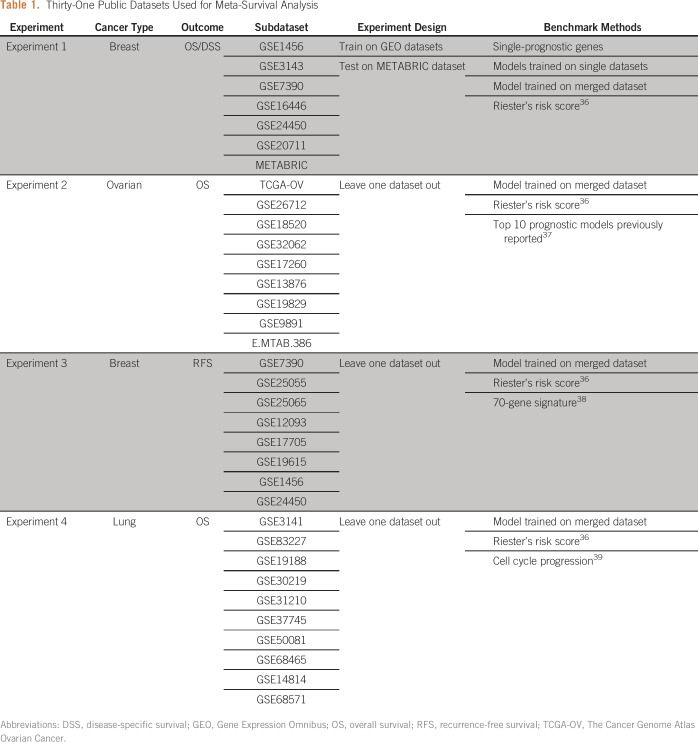
Thirty-One Public Datasets Used for Meta-Survival Analysis

Experiment 1 involved seven microarray datasets of breast cancer with the overall survival (OS) or disease-specific survival information available. We used six GEO datasets for training and the METABRIC dataset for testing. We compared the proposed method with conventional meta-survival analysis methods (Appendix), including single prognostic genes, models trained on a single dataset, and models trained on the merged dataset. It should be noted that for the models trained on a single dataset and on the merged dataset, we also used ridge penalty for regularization so that it would be comparable with the proposed method, which is also regularized by ridge penalty. In addition, we compared it with the meta-analysis method of Riester et al,^36^ which is also able to produce a multigene risk score without merging datasets. Importantly, it estimates the coefficient of each gene independently in a fixed-effect model and thus may be regarded as a marginalized version of the proposed method.

The purpose of experiment 2 was to evaluate the proposed method for predicting OS of high-grade, late-stage ovarian cancer. The benchmark methods included the model trained on the merged dataset, Riester’s risk score, and the top-performing prognostic gene signatures reported in a previous meta-analysis study.^37^ We assessed the prediction performance in a leave-one-dataset-out fashion, where we trained a model on all the datasets except the one that was to be tested. This procedure was repeated until all the datasets were independently tested. It should be noted that in such a process, model training was strictly separated from the test dataset. This means both the gene prioritization and cross-validation–based hyperparameter tuning (λ in ridge regression) were also performed using only datasets allocated for training to avoid overestimation of the result.

Experiments 3 and 4 were designed to compare the performance of meta-survival analysis (ie, the proposed method, the model trained on the merged dataset, and Riester’s risk score) with established multigene signatures in applications where their clinical validity has been extensively tested. In particular, experiment 3 was aimed for prediction of recurrence-free survival (RFS) in eight microarray datasets, for which the 70-gene signature^38^ was implemented. However, experiment 4 concerned OS prediction of non–small-cell lung cancer in 10 microarray datasets, and the CCP signature^39^ was used as the benchmark. For both experiments, signature evaluation was performed in a leave-one-dataset-out manner as described previously.

### Comparison of the Proposed Method With Conventional/Existing Meta-Analysis Methods for Predicting Breast Cancer Disease-Specific Survival

**Fig 2.**
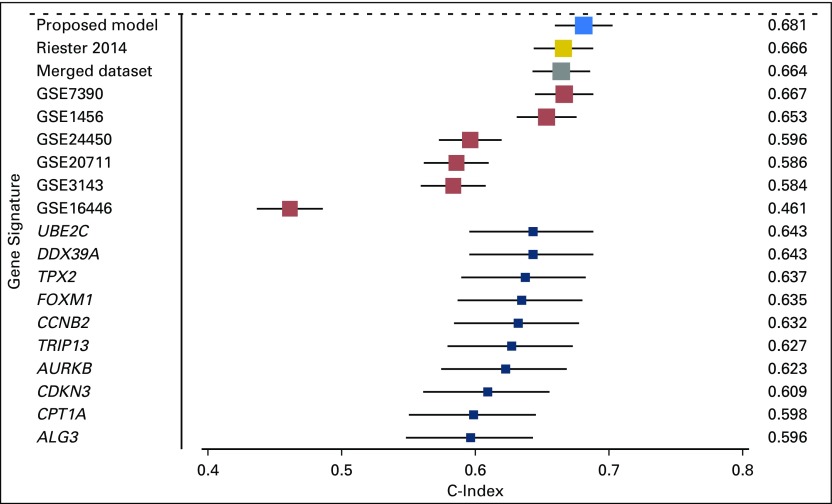
Concordance indices of the proposed decentralized learning method for prediction of breast cancer overall survival on the METABRIC dataset, compared with models trained on single and merged datasets, Riester’s risk score, and single prognostic genes.

In experiment 1, we used the proposed method to train a multigene signature by combining six GEO datasets. When tested on the METABRIC dataset, the resulting signature achieved the highest c-index score of 0.681 (*P* < 2.2 × 10^−16^; *P* values are for Cox regression analysis, unless otherwise indicated) among all competing methods (Fig 2). The Riester’s risk score and the model trained on the merged dataset, respectively, scored a c-index of 0.666 (*P* < 2.2 × 10^−16^) and 0.664 (*P* < 2.2 × 10^−16^) on the METABRIC dataset. We identified single prognostic genes after the meta-analysis approach of Gentles et al.^11^ The top 10 genes in terms of the largest absolute meta-z scores were *CCNB2*, *AURKB*, *TPX2*, *FOXM1*, *TRIP13*, *ALG3*, *CDKN3*, *CPT1A*, *UBE2C*, and *DDX39A*. When evaluated on the METABRIC dataset, the best performing gene was *UBE2C* (c-index, 0.643; *P* < 2.2 × 10^−16^). Finally, we trained gene signatures on each of the six GEO datasets and tested their performances on the METABRIC dataset. The resulting c-index scores showed a wide range, from 0.483 to 0.669.

### Comparison of the Proposed Method With Existing Meta-Analysis Methods and Prognostic Signatures for Predicting Ovarian Cancer OS

**Fig 3.**
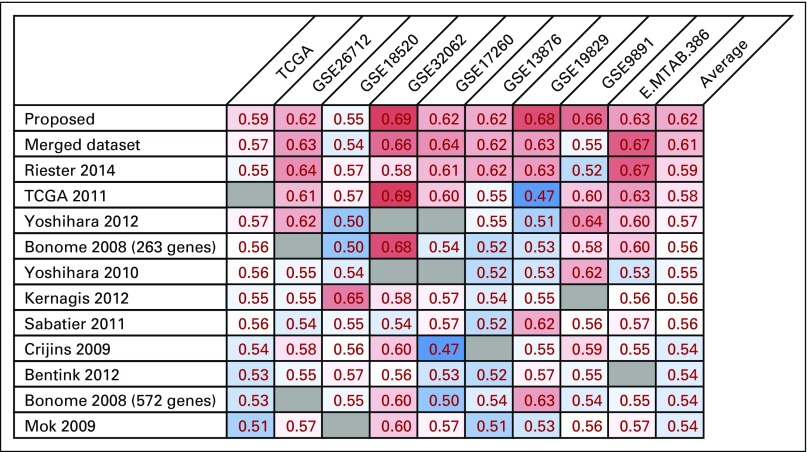
Concordance indices of the proposed method for prediction of ovarian cancer overall survival, compared with the model trained on the merged dataset, Riester’s risk score, and the top 10 prognostic gene signatures previously reported.^37^ Blank cell indicates that the given dataset was used for training the prognostic model in the corresponding row. The average concordance indices were calculated excluding the training datasets. Red cell indicates high C-index. Blue cell indicates low C-index. Gray-shaded cell indicates the corresponding dataset was used for training and therefore its C-index was not reported. TCGA, The Cancer Genome Atlas.

Experiment 2 contained all the datasets used in a previous study by Waldron et al for comparative meta-analysis of prognostic gene signatures for high-grade, late-stage ovarian cancer,^37^ except one dataset because of the retraction of its associated article by Dressman et al.^40^ We used Waldron et al’s code to reproduce the same cohorts in the meta-study. As shown in Figure 3, our gene signature developed using the proposed method had the best average (weighted by sample size) performance (c-index, 0.62) among all competing methods, including the model trained on the merged dataset, Riester’s risk score, and the top 10 prognostic models (which were essentially trained on single datasets) previously reported^37^ (c-index range, 0.55 to 0.61).

### Comparison of the Proposed Method With Existing Meta-Analysis Methods and the 70-Gene Signature for Predicting Breast Cancer RFS

**Fig 4.**
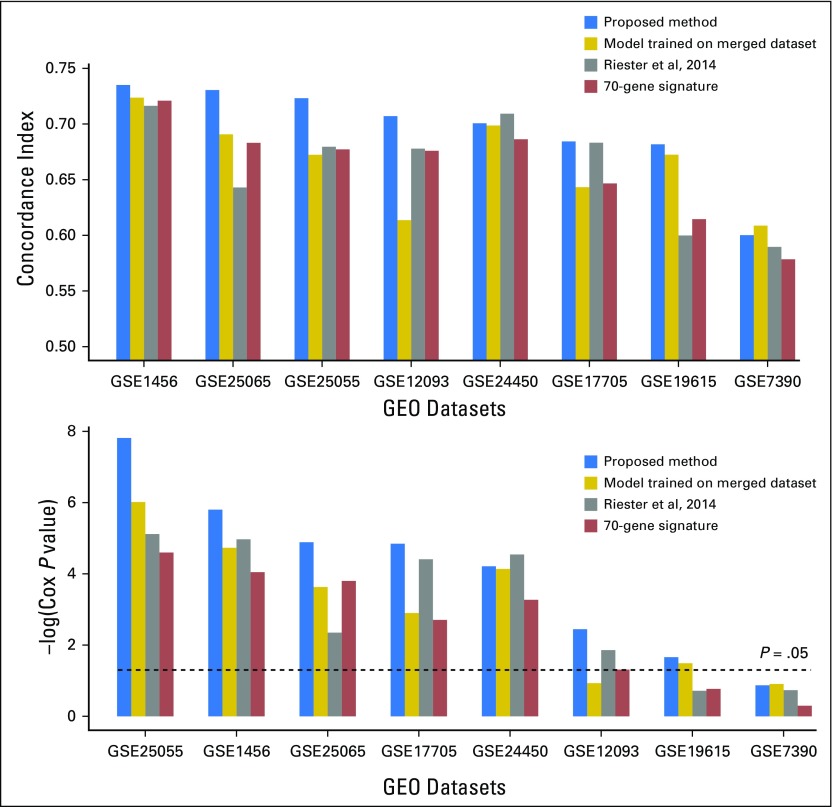
(A) Concordance indices and (B) Cox regression *P* values of the proposed decentralized learning method for prediction of breast cancer recurrence-free survival, compared with the model trained on the merged dataset, Riester’s risk score, and the 70-gene signature. GEO, Gene Expression Omnibus

In experiment 3, the proposed method predicted breast cancer RFS for seven of eight datasets (c-index, 0.682 to 0.735; *P* = .02 to 1.5 × 10^−8^) but not for GEO dataset GSE7390 in leave-one-dataset-out testing. In comparison, the model trained on the merged dataset achieved significant prediction for six datasets (c-index, 0.643 to 0.724; *P* = .03 to 9.7 × 10^−7^) but not for GEO datasets GSE7390 and GSE12093; Riester’s risk score obtained significant results for six datasets (c-index, 0.643 to 0.716; *P* = .01 to 7.6 × 10^−6^) but not for GEO datasets GSE7390 and GSE19615; the 70-gene signature achieved significant prediction for six datasets (c-index, 0.647 to 0.721; *P* = .05 to 2.5 × 10^−5^) but failed for GEO datasets GSE7390 and GSE19615. Overall, the proposed method outperformed the model trained on the merged dataset in seven of the eight datasets, outperformed Riester’s risk score in seven of the eight datasets, and outperformed the 70-gene signature in all the eight datasets in terms of c-index (Fig 4A). Interestingly, the proposed method also outperformed the other three methods in terms of *P* values of Cox regression analysis (Fig 4B), even though it did not explicitly maximize the partial likelihood (as the model trained on the merged dataset does).

### Comparison of the Proposed Method With Existing Survival-Analysis Methods and CCP Signature for Predicting Lung Cancer OS

**Fig 5.**
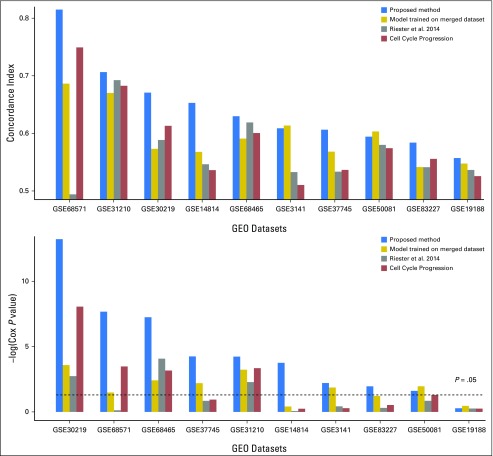
(A) Concordance indices and (B) Cox regression *P* values of the proposed method for prediction of lung cancer overall survival, compared with the model trained on the merged dataset, Riester’s risk score, and the cell cycle progression signature. GEO, Gene Expression Omnibus

In experiment 4, the proposed method predicted lung cancer OS in nine of 10 datasets (c-index, 0.584 to 0.833; *P* = .02 to 6.0 × 10^−14^) in leave-one-dataset-out testing, whereas the result for GEO dataset GSE19188 was not significant. However, the model trained on the merged dataset significantly predicted OS in seven datasets (c-index, 0.568 to 0.686; *P* = .03 to .003) but not for GEO datasets GSE83227, GSE14814, and GSE19188; CCP significantly predicted OS in only four GEO datasets: GSE68465, GSE30219, GSE31210, and GSE68571 (c-index, 0.601 to 0.749; *P* = 6.8 × 10^−4^ to 8.8 × 10^−9^); and Riester’s risk score significantly predicted OS in three GEO datasets: GSE68465, GSE30219, and GSE31210 (c-index, 0.589 to 0.692; *P* = 5.2 × 10^−3^ to 8.4 × 10-^5^). Figure 5 shows that the proposed method consistently outperformed the other three in terms of both c-index and Cox *P* values.

## DISCUSSION

The availability of multitudinous public datasets has provided an opportunity to enhance statistical power and identify more reliable gene signatures by meta-analysis. However, the profound heterogeneity in these data also represents a significant challenge. To address this issue, we proposed a decentralized learning framework for developing robust prognostic signatures on the basis of meta-analysis of multiple gene expression datasets. Through comprehensive evaluation on large-scale datasets totaling more than 6,000 samples of several cancer types, we demonstrated that our method outperformed (1) single prognostic genes identified using conventional meta-analysis, (2) multigene signatures trained on single datasets, (3) multigene signatures trained on merged datasets as well as by existing meta-analysis methods, and (4) clinically applicable, established multigene signatures. These results confirm that the decentralized learning approach can effectively integrate information from multiple datasets and be used to derive robust multigene prognostic signatures.

There are several methodologic advantages of the proposed method that may explain its improved prediction. The decentralized learning approach allows us to perform meta-analysis of multiple datasets without the need for data aggregation. It does this by estimating a common survival model where the loss function is calculated individually on each dataset, and the model coefficients are jointly estimated by minimizing the overall loss function. However, the simple merging approach implicitly assumes that the patients in different cohorts have similar characteristics. In practice, however, significant biologic and clinical differences (eg, cancer stage, histology, or therapies) exist among datasets, which can lead to suboptimal results if data are aggregated without careful selection.

Different from previous studies, we used Harrell’s c-index^41^ as the selection criterion for gene prioritization because it is a direct, more relevant method to measure the survival prediction performance compared with the statistical significance of a Cox regression model. For the same reason, the survival discordance was explicitly used as the loss function for developing gene signatures rather than the pseudo-likelihood of the Cox regression,^13^ the use of which was also proposed by Vikas et al.^15^ There are, however, essential differences between our work and the approach taken by Vikas et al.^15^ First and most important, we integrated such a ranking-based survival model in a decentralized learning framework and therefore successfully addressed the data heterogeneity problem, which to our best knowledge, is the first in meta-survival analysis studies. Second, to better match the ranking nature of the model, we preprocessed the gene expression profiles into population-independent normalized percentile ranks, which served to standardize the data as well as regularize outliers. Third, we used the hinge function to approximate the survival discordance and developed a fast algorithm to evaluate the subgradient for gradient-based optimization. It turned out that both feature transformation and hinge loss approximation can significantly accelerate convergence in model training and improve the model performance.

From a computational perspective, the decentralized learning approach is more efficient because it allows the loss function to be calculated in situ for each dataset. The model training can be accomplished in a distributed fashion, because communication between datasets only involves the transfer of the updated model coefficients and respective losses. This is particularly attractive for large multi-institutional collaborative efforts, such as the CancerLinQ project,^42^ when patient privacy is desired. However, training a model in a merged cohort requires all data to be pooled together in a central database, which demands a huge storage capacity and tremendous computing power.

In principle, our approach can be generalized to incorporate other types of omic data, such as genomic, epigenomic, proteomic, and metabolomic data.^43^ This may help reveal novel molecular mechanisms beyond transcriptome associated with a poor prognosis for patients with cancer. Furthermore, the decentralized learning approach may also be used to identify common biologic themes of aggressive disease across different histologies in a pan-cancer setting.^11^ Currently, our method considers only those common genes across all datasets for building the signature. As the number of datasets increase, this may result in a shrinking of available gene sets. One solution is to impute the missing gene from the common genes.^44^ Although in this study we applied the ridge regularization, which led to a dense model, the *L*^2^-norm can be simply replaced with the *L*^1^-norm or a combination of both^35^ when model sparsity (ie, a small number of genes) is desired.Last but not least, we emphasize that our purpose for applying decentralized learning was to integrate datasets of multiple cohorts presenting the same type of data (eg, gene expression profiles here), whereas the heterogeneity mainly stems from the sampling bias of the population. This should be distinguished from studies that used decentralized learning to integrate multiomics datasets (eg, transcriptomic, proteomic, metabolomic) for the same cohort.^45^ An interesting future study would be to combine these two types of studies to develop more comprehensive models.

In conclusion, we propose a decentralized learning framework for developing multigene prognostic signatures using genome-wide transcriptomic data. Our approach allows us to perform meta-analysis by integrating information from multiple datasets without the need for data aggregation. Given the increasing prevalence of large-scale omic data, this approach can be used to identify robust and more reliable multigene prognostic signatures that will ultimately advance precision medicine. Our codes for implementing the proposed method are publically available at https://github.com/maycuiyan/META-SURV.

## Figures and Tables

**Fig A1.**
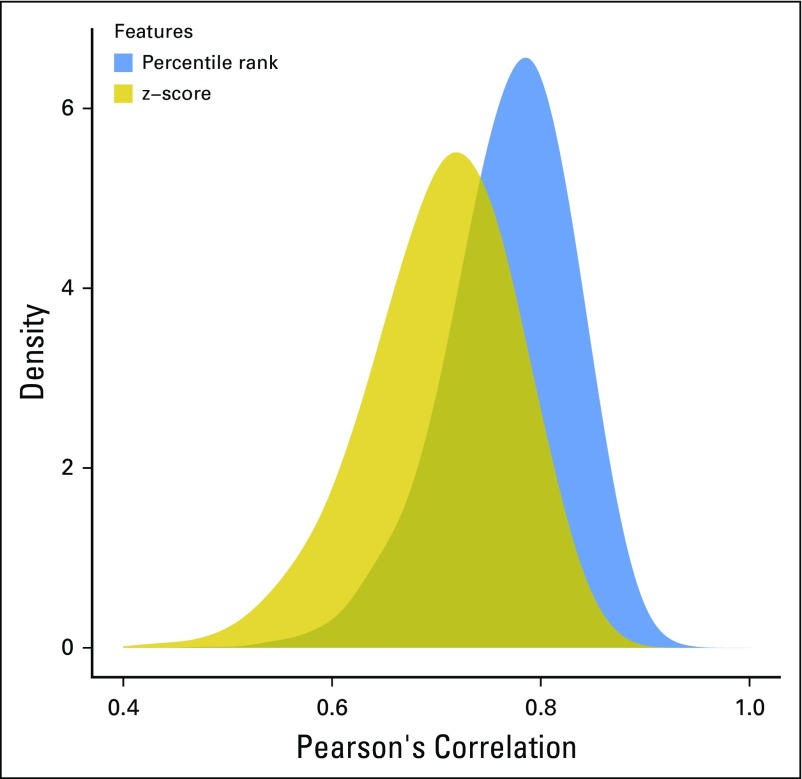
Histograms of sample-wise Pearson correlations between random signatures calculated on microarray and RNA sequencing data using percentile rank and z-score as features.

## Appendix

### Stability of Percentile Rank-Based Gene Signatures

We evaluated the stability of percentile rank-based signatures versus raw expression-based signatures. We used the R package curatedOvarianCancer to obtain both the sequencing and microarray data for the same The Cancer Genome Atlas Ovarian Cancer cohort. We randomly selected 100 genes and obtained two 100-gene signatures, respectively, using the sequencing data and the microarray data. We then computed the sample-wise Pearson correlation to characterize the similarity between the two gene signatures. We found that among 10,000 times of random gene signature generation, the correlation on the basis of percentile ranks ranged between 0.48 and 0.93, showing a higher stability compared with the range of 0.37 to 0.88 on the basis of raw expressions (Appendix Fig A1).

### Conventional Survival-Analysis Methods

Single prognostic genes were identified using the meta-analysis approach of Gentles et al,^11^ whereby genes were prioritized according to the z-score of Cox regression and the Liptak’s weighted meta-z. For single-dataset-trained models, we applied the univariable Cox regression analysis to identify the genes significantly correlated with OS (*P* < .05 with Benjamini-Hochberg [BH] correction, or *P* < .05 without BH correction if the former resulted in an empty list) on the particular dataset. Then these genes were used to train a Cox model with ridge regularization. The penalty strength for ridge regularization was determined by 10-fold crossvalidation.

To train a survival model from the merged dataset, we used ComBat^24^ to correct batch effect for each dataset and merged them together. From the merged dataset, we identified genes significantly correlated with overall survival (*P* < .05 with BH correction) by univariable Cox regression and used them to train a Cox model with ridge regression. Likewise, the penalty strength for ridge regularization was determined by 10-fold cross-validation. We used the R function ComBat in the sva package to implement batch effect correction and the cv.glmnet in the glmnet package to build ridge regularized Cox models.

### Implementation of Established Multigene Signatures

The R package genefu was used to implement the 70-gene signature. The cell cycle progression score was computed by averaging the gene expression values of the 31 genes using the data normalized by the multi-array average algorithm. For GEO datasets GSE83227 and GSE68571, however, only 17 and 15 genes were found present. The cell cycle progression scores for these two datasets were calculated by averaging the available genes, respectively.
